# Factors affecting postdialysis fatigue among hemodialysis patients: a multi-group path analysis according to nutritional status

**DOI:** 10.3389/fmed.2025.1553751

**Published:** 2025-05-13

**Authors:** Yuhan Zhang, Na Yang, Xujiao Bai, Yina Liu, Xiangyun Li, Meiqin Yan, Yuxiang Liu

**Affiliations:** ^1^College of Nursing, Shanxi Medical University, Taiyuan, Shanxi, China; ^2^Department of Nursing, Taiyuan Central Hospital, Taiyuan, Shanxi, China; ^3^Science and Education Section, Shanxi Children’s Hospital, Taiyuan, Shanxi, China; ^4^Department of Nephrology, Shanxi Provincial People’s Hospital, Taiyuan, Shanxi, China; ^5^Department of Nephrology, The Fifth Clinical Medical College of Shanxi Medical University, Taiyuan, Shanxi, China; ^6^Shanxi Provincial Key Laboratory of Kidney Disease, Taiyuan, Shanxi, China

**Keywords:** hemodialysis, postdialysis fatigue, constipation, structural equation model, resilience

## Abstract

**Purpose:**

The aim was to investigate a structural postdialysis fatigue model, to verify the factors which affect the postdialysis fatigue of hemodialysis patients, and to evaluate the moderating effects of nutritional status on postdialysis fatigue through multi-group analysis.

**Methods:**

This multicenter cross-sectional study was conducted in six blood purification centers across hospitals in Taiyuan, Shanxi, China, with a total of 1,281 hemodialysis patients recruited as participants. Data were collected using Pittsburgh Sleep Quality Index, Connor-Davidson Resilience Scale, The Social Support Rate Scale, Post-dialysis fatigue Questionnaire, The Simplified Nutritional Appetite Questionnaire, The Rome IV constipation diagnostic criteria. A multi-group structural equation modeling analysis was then conducted to test the moderating effect of nutritional status on postdialysis fatigue in hemodialysis patients.

**Results:**

The postdialysis fatigue model’s fit indices were adequate. Sleep quality, resilience and social support directly influenced postdialysis fatigue (β = 0.181, −0.322, −0.200, *p* < 0.01). Sleep quality, resilience and social support partial mediated constipation through postdialysis fatigue (β = −0.031, 0.038, 0.024, *p* < 0.01). Multi-group structural equation modeling did support the hypothesis of overall moderation by nutritional status, distinct patterns between the two groups were observed with respect to how background factors and interpersonal triggers related to postdialysis fatigue (Δχ^2^ = 28.879, 155.183, 205.135, 210.135, 230.285, Δdf = 6, 9, 11, 14, 17, *p* < 0.01). The magnitude of the direct effects of sleep quality and resilience on postdialysis fatigue was greater in hemodialysis patients with poor nutritional status (Critical ratios for difference = −1.958, −4.999).

**Conclusion:**

Sleep quality, resilience, social support can directly affect postdialysis fatigue, and can also indirectly affect constipation through postdialysis fatigue. Malnourished hemodialysis patients are more likely to suffer from poor sleep quality, psychological resilience and postdialysis fatigue. Vigorous efforts are needed to improve nutritional status in hemodialysis patients, which ultimately might improve postdialysis fatigue and constipation.

## 1 Introduction

At least one in ten people worldwide lives with kidney disease (1). According to the Global Burden of Disease study, in 2019, over 3.1 million deaths were attributed to kidney dysfunction, making it the seventh leading risk factor for death globally (2). In the United States, Medicare fee-for-service spending for all beneficiaries with chronic kidney disease reached $86.1 billion in 2021, accounting for 22.6% of total expenditures (3). These high global death rates and economic burden reflect disparities in prevention, early detection, diagnosis and treatment of chronic kidney disease. According to Chronic Kidney Disease Collaboration, there are 3.14 million dialysis patients worldwide in 2017 (4). By December 2023, data from the Chinese National Renal Data System showed that the total number of registered hemodialysis (HD) and peritoneal dialysis patients in mainland China had surpassed 1.06 million, with 916,647 HD patients (5). Given the growing number of HD patients and the increasing trend year by year, this chronic patient group demands urgent attention.

Patients with end-stage kidney disease receiving HD suffer a high burden of symptoms after dialysis treatments that adversely impact their quality of life. One of the most prevalent, distressing, and debilitating symptoms is postdialysis fatigue (PDF). Unlike chronic fatigue and intradialytic fatigue, PDF is a unique and debilitating sensation that occurs after dialysis (6). Patients often describe it as feeling “worn out” or “collapsed” (7). Furthermore, the Standardized Outcomes in Nephrology Hemodialysis initiative has identified PDF as a critically important core outcome that must be assessed in HD patients (8).

A meta-analysis involving 12 studies with 1,215 PDF patients found that the overall prevalence of PDF among HD patients was 61.0% (8). PDF exerts multidimensional adverse effects on HD patients. First, severe postdialysis fatigue in HD patients is independently associated with increased all-cause mortality, cardiac mortality, and the composite risk of first cardiac hospitalization (9). Second, studies have demonstrated that HD patients with PDF are more likely to experience decreased daily activities, weakened ability to maintain interpersonal relationships, and failure to meet others’ expectations. Lastly, a study highlights the particularly high incidence of depression, anxiety, and cognitive impairment in HD patients with PDF (10). Given these significant impacts, it is critical to effectively identify the risk factors and pathways underlying the occurrence of PDF.

Although previous studies have identified factors affecting PDF, there is still a need for more comprehensive understanding. Since most HD patients simultaneously face multiple factors influencing PDF, evaluating it across various contexts is crucial. Research that identifies structural relationships among variables with direct and indirect effects on PDF can provide valuable insights for designing effective nursing care programs for HD patients with PDF. At the same time, whether constipation is a symptom of fatigue after dialysis remains to be explored. Furthermore, a review of existing literature reveals no studies that explore the path relationships between risk factors, PDF, nutritional status and constipation in HD patients.

Studies have found that at least one-third to one-half of dialysis patients have malnutrition problems (11). According to the American Society for Parenteral and Enteral Nutrition, malnutrition refers to all deviations from adequate and optimal nutritional status, including energy undernutrition and overnutrition (12). The term “undernutrition” is used to refer to generally poor nutritional status; however, as malnutrition often refers to undernutrition, these terms are widely used in a similar sense (13). As the main factor affecting the survival rate and prognosis of HD patients, poor nutritional status can also lead to fatigue after dialysis. Antonio found that higher protein intake is independently associated with lower risk of moderate and severe fatigue (14). On the contrary, Subrata found that fatigue (measured by severity and interference) was more obvious on dialysis days than on non-dialysis days, but these differences could not be explained by nutritional status (15). In conclusion, previous studies have mainly investigated the relationship between nutritional status and fatigue, but no research has reported the degree of influence on the path to PDF according to nutritional status, despite nutritional status being a modifiable variable.

In this regard, the present study aimed to investigate a structural model exploring the factors directly and indirectly affecting PDF and constipation, and to evaluate nutritional status moderating effects on PDF and constipation through multi-group (well-nourished vs. malnourished) analysis in HD patients.

## 2 Materials and methods

### 2.1 Conceptual framework of the study

This study developed a conceptual framework to analyze and validate the associations between factors such as sleep quality, resilience, and social support that influence PDF and its performance constipation among HD patients. According to the Theory of Unpleasant Symptoms (16), this theoretical model describes the factors influencing symptom experiences and explains the mechanisms of symptom generation. Theory of Unpleasant Symptoms framework comprises three core concepts: symptoms, influencing factors, and outcome performance. Influencing factors are categorized into physiological, psychological, and situational domains, while symptom performance is multidimensional. These elements are interdependent and mutually constraining, collectively influencing an individual’s overall health and wellbeing.

**FIGURE 1:**
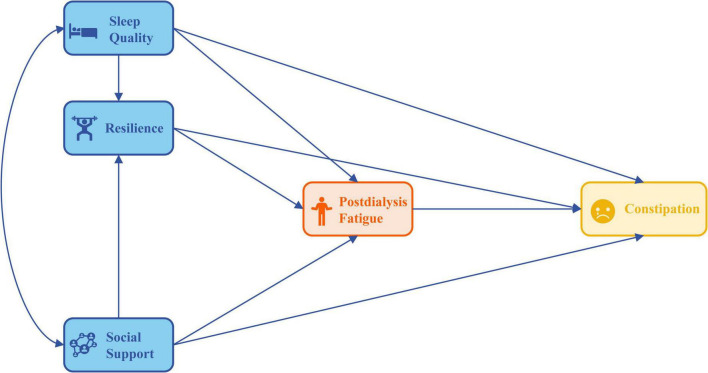
Hypothesized model of the study.

The experience of PDF presents significant challenges and demands for HD patients, resulting in unpleasant symptoms and diminished quality of life (17). According to Theory of Unpleasant Symptoms, sleep quality is categorized as a physiological factor, resilience as a psychological factor, social support as an environmental factor, PDF as unpleasant symptoms and constipation as its performance. These factors shape how HD patients interpret and cope with their unpleasant symptoms, such as PDF, which is critical in determining their stress responses (18, 19). The study’s model proposed that sleep quality, resilience, and social support directly affect the levels of PDF and constipation in HD patients. Additionally, the model recognized that these influencing factors can also indirectly impact constipation through their effects on PDF. Nutritional status was classified as a moderating factor affecting all paths. Figure 1 illustrates the theoretical model developed in this study.

### 2.2 Study design and participants

A descriptive, multicenter, cross-sectional study was conducted with ethical approval granted by the Ethics Committee of the Fifth Clinical Medical College of Shanxi Medical University (Approval No. SPPH-2024-424). All methods adhered to the Declaration of Helsinki. The study findings were reported in compliance with the Strengthening the Reporting of Observational Studies in Epidemiology (STROBE) guidelines.

The sampling process in this study involved two stages. First, three districts were randomly selected from six districts in Taiyuan City, Shanxi Province. Next, HD patients were recruited using convenience sampling from the designated blood purification centers in hospitals within these districts. The selected hospitals included Shanxi Provincial People’s Hospital, the First Hospital of Shanxi Medical University, and Shanxi Provincial Hospital of Traditional Chinese Medicine in Yingze District; the Second Hospital of Shanxi Medical University and Taiyuan Central Hospital in Xinghualing District; and Shanxi Bethune Hospital and Taiyuan Central Hospital in Xiaodian District.

HD patients meeting the inclusion and exclusion criteria were recruited between May 2024 and October 2024. The inclusion criteria were as follows: ① age ≥ 18 years; ② initial regular hemodialysis treatment ≥ 3 months, at least 2 times a week; ③ consciousness is clear, communication and cognitive ability is normal; ④ informed consent, voluntary participation. The exclusion criteria included meeting any of the following conditions: with severe cardiovascular disease, severe infection, cancer and other important organ diseases; chronic fatigue state; pregnant women.

### 2.3 Measures

Participants provided information on their age, gender, BMI, marital status (categorized as married, unmarried, divorced or widowed), occupation status (categorized as full time job, part time job, no job), education level (categorized as high school or less, college or above), whether to eat during dialysis treatment. A history and physical examination can diagnose functional constipation using the Rome IV diagnostic criteria (20).

The Pittsburgh Sleep Quality Index (PSQI), developed by Buysse et al. (21), is primarily used to evaluate sleep situation over the past month. The PSQI comprises 19 self-rated items grouped into seven components: subjective sleep quality, sleep latency, sleep duration, habitual sleep efficiency, sleep disturbances, use of sleeping medication, and daytime dysfunction. Each component score ranges from 0 to 3, and higher scores indicate worse sleep quality. The total PSQI score is obtained by summing the component scores, with a range of 0 to 21, and the higher the score, the worse the sleep quality. It has been applied in HD patients, with a Cronbach’s α coefficient of 0.789.

The Connor Davidson Resilience Scale (CD-RISC-10) (22) was adapted by Connor. The scale is a single-dimensional scale to assess the patient’s psychological resilience level. There are 10 items in the scale, and the Likert 5-level scoring method (0 = never, 1 = rarely, 2 = sometimes, 3 = often, 4 = always) is used to score 0–40 points. The higher the total score, the higher the level of resilience. The Cronbach’s α coefficient of this study was 0.950 in HD patients.

The Simplified Nutritional Appetite Questionnaire (SNAQ) is a tool designed by The Council for Nutritional Strategies in Long-Term Care to assess appetite and nutritional status for patients with chronic illnesses (23). As a simplified version of Nutrition Appetite Questionnaire (CNAQ), SNAQ can quickly and effectively identify patients with decreased appetite, which may lead to malnutrition and related complications. The SNAQ consists of four key questions that generally cover the following aspects: Changes in appetite over a specific period; Overall interest in food; Preferences or aversions to specific foods; Changes in eating habits. Each question is typically rated on a 5-point scale, SNAQ score ≤ 14 indicates significant risk of at least 5% weight loss within six months.

The Social Support Rate Scale (SSRS), developed by Xiao (24), is a tool designed to measure the level of social support an individual receives. The SSRS consists of 10 items, evaluating different dimensions of social support, including emotional support, instrumental support, and informational support. The total score is 66, with higher scores indicating greater social support. 0–22 indicates low social support, 23–44 indicates moderate social support, and 45–66 indicates high social support. In HD patients, with a Cronbach’s α coefficient of 0.769.

The assessment of PDF was conducted according to the recommendations by Sklar et al. (25). Patients were classified as experiencing PDF if they spontaneously reported feeling fatigued in response to the open-ended question: “Do you feel fatigued after dialysis?” If the response was affirmative, each patient rated the intensity, duration, and frequency of their fatigue on a scale from 1 to 5. Intensity was defined as the magnitude of fatigue, duration as the length of time that fatigue lasted, and frequency as the number of times that fatigue happened. PDF exists when the total score of the three indicators is ≥ 4.

### 2.4 Data collection

The researchers conducted one-on-one interviews to administer the questionnaire. Before distributing the questionnaire, they provided participants with a detailed explanation of the study’s purpose, procedures, potential benefits, and risks. Only after ensuring that the HD patients had a full understanding and willingly agreed to participate, they were asked to sign the informed consent form. Before beginning the data collection, a preliminary pilot test was conducted with seven HD patients to assess the comprehensibility of the questionnaire. Based on feedback from the pilot test, necessary modifications were made to the questionnaire. To express gratitude, all participants who completed the questionnaire received a small gift. Among the gifts, soap is used to clean autogenous arteriovenous fistula, medicine bag is used to store and carry daily medicine for HD patients, the bandage is used to stop bleeding after the needle is pulled out after dialysis.

### 2.5 Statistical analysis

Data analysis was performed using IBM SPSS version 26. Descriptive statistics were used to summarize demographic variables, such as age, gender, marital status, occupation states, education level and weather to eat during dialysis and constipation with results presented as frequencies and percentages. Pearson’s correlations were used to explore the associations between sleep quality, resilience, social support, PDF and constipation. Regarding the handling of missing data, we remove observations with missing values greater than 20% during the data cleaning stage and apply the KNN algorithm for imputation; Then full information maximum likelihood is often preferred for SEM (26). Structural equation modeling (SEM) was conducted using R version 4.4.0 with the “lavaan” package (27). Path analysis was used to identify both direct and indirect relationships in the model. Standardized regression coefficients (β) and *P* values for β were reported for direct and indirect effects. The model fit was assessed using the following model-fit indices: relative chi-square (χ^2^/df) test < 2 (*p* > 0.05), goodness of fit index (GFI) > 0.90, adjusted goodness of fit index (AGFI) > 0.90, comparative fit index (CFI) > 0.90, normed fit index (NFI) > 0.90, incremental fit index (IFI) > 0.90, Tucker–Lewis index (TLI) > 0.90, and root mean squared error of approximation (RMSEA) < 0.06 (28). The Akaike information criterion (AIC) was applied to compare the modified model with the initial model. Bias-corrected intervals were used in this study, and the number of bootstrap samples was set to 1,000.

For the present study, using the final base structural equation model, a multi-group structural equation modeling was then conducted to examine the moderation effect of nutritional status on the pathways by testing the invariance of the pathways across the two groups (Well-nourished vs. Malnourished group). In the multi-group structural equation modeling, equality constraints were imposed on the pathways in the constrained model, and the data for both groups were simultaneously analyzed to obtain efficient estimates. In the unconstrained model, the pathways were allowed to vary across the two groups. The nested χ^2^ statistic was used to compare the fit between constrained and unconstrained models. The criterion for determining presence of a moderation effect was as follows: If the strengths of pathways among the variables in the model were statistically significantly different between two groups, then a moderation effect existed (29). For the significance of path differences between groups, “critical ratios for differences” were used (30). Holm-Bonferroni correction method were used to control Type I error rates. Meanwhile, configural/metric/scalar invariance were tested. The statistical significance of direct and indirect effects was tested using the bootstrap resampling method with 1,000 resamples. *P*-value < 0.05 (two-sided) was considered statistically significant.

## 3 Results

### 3.1 Demographic and clinical characteristics of the participants

The general characteristics of the 1,281 subjects are presented in Table 1. The majority of whom were male (65.3%) and between 18 and 59 years old (62.8%). A total of 1,158 individuals (90.4%) were married. The educational level of the majority of older adults was high school or less, accounting for 83.9% (*n* = 1,074), while 207 individuals (16.1%) had completed college or above. The BMI levels of most patients were in the normal range (55.7%). In terms of employment status, 86.8% (*n* = 1,112) of the HD patients were unemployed, including retired individuals. 71% of HD patients had eating behavior during dialysis; 35.7% of HD patients had constipation symptoms. The number of subjects in the well-nourished group as determined by the SNAQ was 712 (55.6%), while 569 (44.4%) subjects were determined to be at risk of malnutrition or in the malnourished group.

**TABLE 1:** Distribution of demographic characteristics of hemodialysis patients and scales points.

Variables	Total (*n* = 1,281)	Malnourished group (*n* = 569)	Well-nourished group (*n* = 712)	*p*-value
**Age,% (*n*)**				0.919
< 60	62.76 (804)	62.85 (358)	62.69 (446)	
≥ 60	37.24 (477)	37.06 (211)	37.34 (266)	
**Gender,% (*n*)**				0.070
Male	65.3 (836)	62.57 (356)	67.50 (480)	
Female	34.7 (445)	37.43 (213)	32.56 (232)	
**BMI,% (*n*)**				< 0.001
< 18.5	9.8 (126)	10.37 (59)	9.40 (67)	
18.5–23.9	55.7 (714)	62.62 (357)	47.60 (339)	
24–27.9	24.8 (318)	18.62 (106)	29.76 (212)	
≥ 28	9.60 (123)	5.10 (29)	13.21 (94)	
**Marital status,% (*n*)**				0.451
Married	90.4 (1,158)	89.92 (512)	91.73 (646)	
Unmarried	6.60 (85)	6.49 (37)	6.74 (48)	
Divorced	2.20 (28)	2.28 (13)	2.11 (15)	
Widowed	0.80 (10)	1.23 (7)	0.42 (3)	
**Occupation status, % (*n*)**				< 0.001
Full time job	8.40 (108)	6.15 (35)	10.26 (73)	
Part time job	4.80 (61)	2.11 (12)	6.88 (49)	
No job	86.8 (1,112)	91.74 (522)	82.92 (590)	
**Educational level, % (*n*)**				< 0.001
High School or less	83.91 (1,074)	87.96 (501)	80.56 (573)	
College or above	16.16 (207)	11.96 (68)	19.51 (139)	
**Eat during dialysis, % (*n*)**				0.254
Yes	71.0 (910)	69.38 (395)	72.31 (515)	
No	29.0 (371)	30.62 (174)	27.65 (197)	
**Constipation, % (*n*)**				< 0.001
Yes	35.7 (457)	41.14 (234)	31.25 (223)	
No	64.3 (825)	58.84 (335)	68.62 (489)	
PSQI, mean (SD)	8.64 (3.45)	9.36 (3.24)	8.06 (3.51)	< 0.001
CD-RISC, mean (SD)	25.27 (6.79)	22.80 (6.59)	27.25 (6.28)	< 0.001
SSRS, mean (SD)	25.52 (5.10)	24.95 (4.91)	25.98 (5.19)	< 0.001
PDF, mean (SD)	5.25 (4.22)	6.22 (3.65)	4.48 (4.47)	< 0.001

BMI, body mass index; PSQI, Pittsburgh Sleep Quality Index; CD-RISC, Connor-Davidson Resilience Scale; SSRS, Social Support Rate Scale; PDF, post-dialysis fatigue; *p*-value was presented for chi-square tests for categorical variables and for *t*-test for continuous variables.

### 3.2 Associations between the study variables

Descriptive statistics and correlation analyses for the study variables are presented in Tables 1, 2. The mean scores of sleep quality (PSQI), resilience (CD-RISC), social support (SSRS), PDF were 8.64 (SD = 3.45), 25.27 (SD = 6.79), 25.52 (SD = 5.10) and 5.25 (SD = 4.22), which were approximately 41.1, 63.17, 38.7 and 35.0% of the total score, respectively. Pearson analysis showed that all variables were correlated (*r* = −0.072 to 0.306, *p* < 0.05).

**TABLE 2:** Correlation coefficients among variables (*n* = 1,281).

Variables	Correlations between the scales				
	1	2	3	4	5
PDF (1)	1.00				
PSQI (2)	0.306**	1.00			
CD-RISC (3)	−0.384**	−0.234**	1.00		
SSRS (4)	−0.279**	−0.250**	0.107**	1.00	
Constipation (5)	−0.152**	−0.072*	0.067*	0.150**	1.00

*p < 0.05.

**p < 0.01.

### 3.3 Path analysis

**FIGURE 2:**
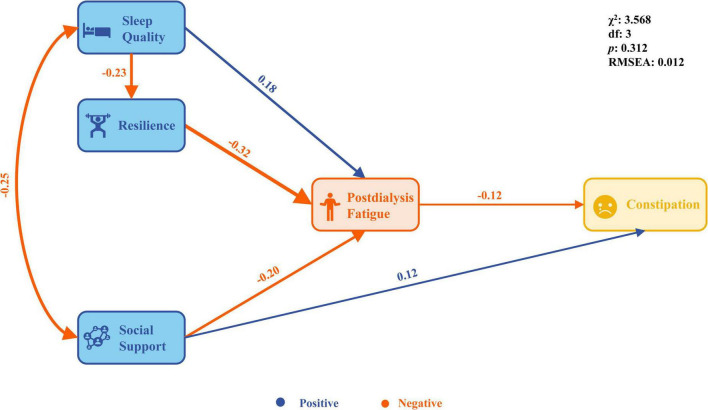
The final model.

Based on the results of Pearson’s correlation analysis, the Theory of Unpleasant Symptoms, and relevant professional knowledge, a structural equation model of influencing factors of PDF and constipation was constructed. On the basis of the hypothesis model (saturation model), we deleted the path without statistical significance and found that the fitting indexes performed well (χ^2^ = 3.568, df = 3, *p* = 0.312, χ^2^/df=̃ 1.189, NFI=̃ 0.993, CFI=̃ 0.999, TLI=̃ 0.996, RMSEA = 0.012, AIC = 37.568 and ECVI=̃ 0.029, Table 3). The final model results show that sleep quality was directly associated with resilience (β = −0.23, *p* = 0.003); sleep quality, resilience and social support was directly associated PDF (β = 0.18, −0.32, −0.20, *p* < 0.01); social support and PDF was directly associated constipation (β = 0.12, −0.12, *p* < 0.01); sleep quality can indirectly affect PDF through resilience (β = 0.075, *p* < 0.01); sleep quality, resilience and social support can indirectly affect constipation through PDF (β = −0.031, 0.038, 0.024, *p* < 0.01, Figure 2).

**TABLE 3:** Fit index values of basic model and multiple group comparisons.

Model	CMIN	DF	*P*	CMIN/DF	NFI	RFI	IFI	TLI	CFI	RMSEA	AIC	ECVI
Hypothetical model	0	0	–	–	1	–	1	–	1	0.205	40	0.031
Final model	3.568	3	0.312	1.189	0.993	0.978	0.999	0.996	0.999	0.012	37.568	0.029
Unconstrained	7.249	6	0.298	1.208	0.985	0.949	0.997	0.991	0.997	0.013	75.249	0.059
Structural weights	36.128	12	0.000	3.015	0.924	0.873	0.948	0.911	0.947	0.04	92.185	0.072
Structural intercepts	162.432	15	0.000	10.829	0.658	0.544	0.679	0.568	0.676	0.088	212.432	0.166
Structural means	212.384	17	0.000	12.496	0.553	0.474	0.573	0.495	0.57	0.095	258.43	0.202
Structural covariances	217.384	20	0.000	10.869	0.542	0.542	0.566	0.566	0.566	0.088	257.384	0.201
Structural residuals	237.534	23	0.000	10.328	0.500	0.565	0.525	0.59	0.528	0.085	271.534	0.212

CMIN, chi-square minimum; DF, degrees of freedom; NFI, normed fit index; RFI, relative fit index; IFI, incremental fit index; TLI, Tucker–Lewis index; CFI, comparative fit index; RMSEA, root mean square error of approximation; AIC, Akaike information criterion; ECVI, expected cross-validation index.

### 3.4 Multi-group analysis

**FIGURE 3:**
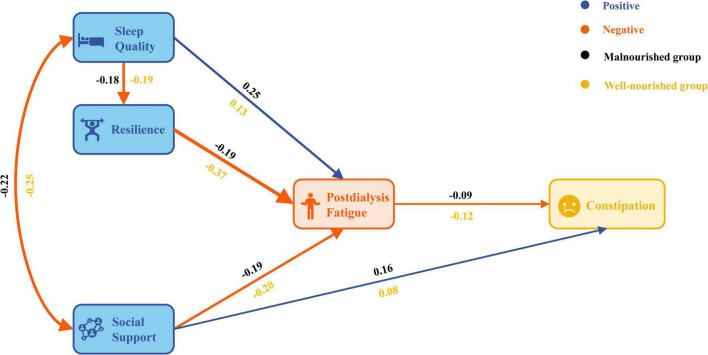
PDF path diagram according to nutritional status.

A multi-group structural equation modeling was conducted to test the moderation effect of nutritional status on the hypothesized paths (Figure 3). The results showed that the fitting indexes of the unconstrained model were within the acceptable range and could be compared across groups (χ^2^ = 7.249, df = 6, *p* = 0.298, χ^2^/df = 1.208, NFI = 0.985, CFI = 0.997, TLI = 0.991, RMSEA = 0.013, AIC = 75.249 and ECVI = 0.059). The unconstrained and constrained models were statistically different (Δχ^2^ = 28.879, 155.183, 205.135, 210.135, 230.285, Δdf = 6, 9, 11, 14, 17, *p* < 0.01, Table 4). The results of Holm-Bonferroni correction show that the *p*-values of all five Δχ^2^ tests remain significant, as all the corrected *p*-values are less than 0.05. The configural and metric tests were successfully passed, but the results of the scalar invariance test indicated a *p*-value < 0.001. However, since the main aim of this study is to compare the relationship structure between groups rather than the latent variable scores, scalar invariance may not be as crucial for our analysis. The malnourished group paths of sleep quality to PDF (critical ratios for difference = −1.958) and resilience to PDF (critical ratios for difference = −4.999) were determined to be significantly different when compared with the well-nourished group (Table 5).

**TABLE 4:** Comparison between unconstrained and constrained model.

Model	Δ CMIN	Δ DF	*P*	Δ NFI	Δ RFI	Δ IFI	Δ TLI	Δ CFI
Structural weights	28.879	6	< 0.01	−0.061	−0.076	−0.049	−0.08	−0.05
Structural intercepts	155.183	9	< 0.01	−0.327	−0.405	−0.318	−0.423	−0.321
Structural means	205.135	11	< 0.01	−0.432	−0.475	−0.424	−0.496	−0.427
Structural covariances	210.135	14	< 0.01	−0.443	−0.407	−0.431	−0.425	−0.431
Structural residuals	230.285	17	< 0.01	−0.485	−0.384	−0.472	−0.401	−0.469

**TABLE 5:** Parameter estimates of variables for the basic model and models according to nutritional groups.

Path	Complete				Male				Well				Critical ratios for difference
	β	Lower	Upper	*p*	β	Lower	Upper	*p*	β	Lower	Upper	*p*	
**Direct effects**													
Sleep quality–resilience	−0.234	−0.288	−0.174	0.003	−0.183	−0.267	−0.095	0.002	−0.191	−0.267	−0.108	0.002	0.306
Sleep quality–PDF	0.181	0.127	0.234	0.002	0.253	0.161	0.341	0.003	0.129	0.063	0.200	0.001	−1.958*
Social support–PDF	−0.200	−0.252	−0.148	0.002	−0.194	−0.277	−0.118	0.002	−0.198	−0.262	−0.127	0.003	−0.633
Social support–constipation	0.118	0.067	0.172	0.001	0.158	0.074	0.242	0.003	0.08	0.005	0.162	0.036	−1.574
Resilience-PDF	−0.322	−0.373	−0.274	0.001	−0.188	−0.274	−0.108	0.001	−0.372	−0.442	−0.31	0.002	−4.999*
PDF–constipation	−0.119	−0.171	−0.062	0.002	0.088	−0.169	−0.005	0.038	−0.119	−0.192	−0.042	0.005	−0.084
**Indirect effects**													
Sleep quality–PDF	0.075	0.054	0.097	0.002	0.034	0.015	0.063	0.001	0.071	0.041	0.107	0.002	
Sleep quality–constipation	−0.031	−0.048	−0.015	0.002	−0.025	−0.054	−0.002	0.029	−0.024	−0.045	−0.009	0.003	
Social support–constipation	0.024	0.013	0.038	0.001	0.017	0.002	0.039	0.026	0.024	0.009	0.042	0.003	
Resilience–constipation	0.038	0.021	0.058	0.002	0.016	0.002	0.004	0.022	0.044	0.017	0.074	0.004	

*p < 0.05.

## 4 Discussion

The results of our multicenter survey indicate a high incidence of PDF and constipation symptoms among HD patients. This study also highlights the interrelationships among sleep quality, resilience, social support, PDF, and constipation and provides understanding of the factors that may potentially improve patient outcomes. By and large, the structural model illustrates that sleep quality, resilience and social support directly associated with PDF and can affect constipation indirectly through PDF. The results of multi-group analysis showed that nutritional status was a moderating variable in the overall structural equation model. These findings emphasize the importance of addressing PDF symptoms in HD patients by considering physiological, psychological, and environmental factors in nursing care.

Our survey revealed an incidence rate of PDF of 0.62. The present study’s result is close to a meta-analysis’s result 0.61 which included 12 studies and 2,152 HD patients (31). Other research has found that prevalence estimates of PDF range from 20% to 86% (32). Variations are likely due to differences in inclusion criteria, assessment methods, or definitions of fatigue used across studies. There is currently no consensus on the definition or measurement of PDF. One of the more consistently used measures quantifies PDF by quantify duration, frequency, and intensity of fatigue on 5-point Likert scales (25). Another used measure is time to recovery from dialysis (TIRD), a validated indirect measure of PDF in which patients report the time required to recover from an HD session (33). Since PDF is different from chronic fatigue and intradialytic fatigue, it remains to be further explored whether it is appropriate to use a universal fatigue measurement tool that is not aimed at HD patients. In conclusion, the high incidence of PDF in HD patients significantly limits their participation in daily life, increases the incidence of adverse events, and increases psychological cognitive obstacles. Therefore, how to accurately identify the risk factors of PDF and accurately intervene to improve the symptoms of PDF needs urgent attention.

Our multicenter survey found that the incidence of constipation in HD patients was 0.36. This is close to the results of 0.38 by Bulbul et al. (34). The causes of constipation in HD patients may be reduced water intake, reduced intake of high-fiber food, less daily activity, neurological lesions, and drug side effects lead to constipation in HD patients. At the same time, intestinal flora imbalance may also be the cause of constipation in HD patients. Because of the accumulation of a large number of metabolites in the blood of dialysis patients, it can flow into the intestinal tract in severe cases, change the intestinal environment, lead to intestinal flora imbalance, and further cause constipation. The results of Zhang’s study suggest that there is a difference in the intestinal flora between maintenance HD patients with constipation and maintenance HD patients without constipation (35). As a known risk factor for chronic kidney disease and end-stage renal disease, constipation is also a common complication of HD patients, which seriously affects their quality of life. Recently, the results of the Seattle Institute of Systems Biology showed that constipation was negatively correlated with liver and renal function. One of the microbial metabolites, toxin 3-indoxyl sulfate (3-IS), mediates the effect of bowel movement frequency on renal function (36). Therefore, fecal microbiota transplantation and probiotics maybe feasible treatments for functional constipation, but there is still a gap in the research on the characteristics of gut flora in patients with maintenance HD combined with constipation.

The study identified different pathways of relationships among the variables. These results underscore the significant role of sleep quality, resilience, and social support as physiological, psychological, and environmental factors in mitigating PDF symptoms of HD patients. Based on the Theory of Unpleasant Symptoms and present path analysis results, we preliminarily propose that PDF in HD patients can be categorized into four subtypes, namely physiological type, psychological type, environmental type and mixed type. In light of this, within the framework of modern scientific and precise healthcare (37), according to the different type of PDF, nursing should be carried out according to different clinical phenotypes. At the same time, because post-dialysis fatigue is different from inter-dialysis fatigue and chronic fatigue, the development of targeted measurement tools for dialysis patients should include the above three dimensions. In addition to the direct effect, the three indirect effects are all caused by fatigue after dialysis. Therefore, whether PDF is the core symptom in the symptom group of HD patients and whether PDF plays a role as a core bridge in the symptom group needs further exploration.

In terms of the relationship between nutritional status and PDF, the poorer the nutritional status, the higher the dialysis recovery time. According to a cross-sectional study on the relationship between dialysis recovery time and malnutrition among end-stage renal disease patients on regular HD, the risk ratio of malnutrition for PDF was 21.69 (38). The present study evaluated nutritional status as a moderating effect on PDF in HD patients. Our findings support and contribute to previous studies reporting that PDF in HD is associated with multidisciplinary factors, and that poor nutritional status causes a decrease in sleep quality, resilience and social support leading to constipation. Furthermore, in the present study, poor nutritional status accelerated PDF by nearly two times when compared with the well-nourished group, as sleep quality decreased. In addition, as the patient’s resilience decreased, PDF were increased two times more in the malnourished group when compared with the well-nourished group. Nutritional status is a variable that can be targeted through nursing interventions, though this was never mentioned in other studies that evaluated the moderating effects of nutritional status on the PDF path.

Our findings have several practical implications for nurses, healthcare providers, and researchers involved with HD patients care. First of all, from the perspective of precision nursing science, medical staff should accurately identify different subtypes of fatigue after dialysis and carry out targeted nursing on this basis. Secondly, PDF is very common and harmful in HD patients, and subsequent research can use the potential category analysis method to classify PDF deeply. At the same time, symptom network analysis can verify whether PDF is the core symptom in the symptom group of HD patients and whether it has the main bridge function. Lastly, poor nutritional status significantly decreases sleep quality, lowered psychological resilience, and increased incidence of constipation and PDF. Based on these results, to improve interventions on and prevent constipation and PDF in HD patients, more attention should be paid to improving nutritional status.

### 4.1 Limitations

This study is one of the few that examines the factors influencing PDF and constipation using a multi-group structural equation model. However, several limitations should be noted. First, although structural equation model is a powerful tool for this purpose, the cross-sectional design may introduce result bias. Longitudinal research is needed to determine causal relationships among the study variables. Additionally, this study was conducted exclusively in a Chinese population, and the generalizability of the findings to other populations should be interpreted with caution. Future research should carry out international surveys to strengthen its generalization. Furthermore, the use of self-report questionnaires in this study may have introduced biases, such as social desirability, recall, and reporting bias. Finally, the path coefficient of sleep quality affecting PDF is marginally significant in both groups, requiring careful interpretation. Subsequent high-quality studies are needed for verification. However, the present study did represent the real situation of PDF in HD patients that PDF is a common symptom.

## 5 Conclusion

In conclusion, sleep quality, resilience and social support were important factors that directly affected PDF. Furthermore, The above three factors can further affect constipation through PDF. At the same time, nutritional status plays a moderating effect in the whole model. Findings from this study should alert clinicians and nurses to take into account the presence of nutritional status when planning care for HD patients.

## Data Availability

The raw data supporting the conclusions of this article will be made available by the authors, without undue reservation.
